# Cervical spine injury in children: a case report and literature review

**DOI:** 10.11604/pamj.2015.20.261.6071

**Published:** 2015-03-18

**Authors:** Tahir Nebhani, Hicham Bakkali, Lahcen Belyamani

**Affiliations:** 1 Emergency Department of Military Hospital, Rabat, Morocco

**Keywords:** cervical, spine, injury, children

## Abstract

Traumatic injuries of the cervical spine are less common in children than in adults. But may be associated with significant disability and mortality. Pediatric victims of blunt trauma have mechanisms of injury, developmental and anatomic characteristics different than the adults. The purpose of this observation is to highlight the differences between the adult and pediatric cervical spine. We report below the case of spinal cord cut occurs to a very young girl after a motor vehicle accident.

## Introduction

Cervical spine injury is uncommon in children than in adults, with 1-2 percent of pediatric trauma victims requiring hospitalization [1]. However cord injury may have devastating consequences. In fact, I have, through the ensuing of this case, determine the epidemiology, risk factors, mechanisms, levels, types of injury and comorbid factors associated with these potentially devastating injuries.

## Patient and observation

**Figure 1:**
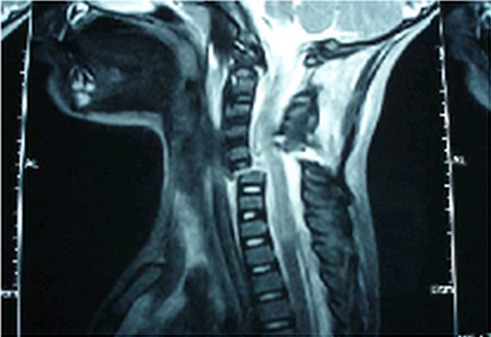
Medullary full section on a sagittal section of MRI of the cervical spine

We report a case of a 3-year-girl, victim of a car accident. Rear passenger seat on the arms of his mother. One hour later she was admitted in a pediatric emergencies department. Her neck was immobilized by a collar. She was conscious, limited neck movement with central pain and she presented a full tetraplegia with loss of feeling and movement from the chest down, including both arms and both legs. There was an unexplained hypotension with heart rate irregularity, breathing difficulties. The rest of the clinical examination was without particularity. The patient was correctly immobilized in neutral position. She was intubated and ventilated. A mean arterial pressure of 85mmHg was obtained by use of dopamine in electric syringe pump at a dose of 7 ug / kg / min. Hematological investigations (include RBC count, hemoglobin concentration and hematocrit) were normal. The magnetic resonance imaging demonstrated a complete cervical medullary section at C6-C7 level (Figure 1). The patient did not recive methylprednisolne, and neither spinal traction nor surgical decompression had been achieved. The Evolution was fatal after 10 days of hospitalization in the pediatric intensive care unit with nosocomial pulmonary infection complicated by a refractory septic shock.

## Discussion

Cervical spine (C-spine) injuries occur infrequently in children but may be associated with significant disability and mortality [2]. It is seen primarily in those who sustain significant, severe blunt trauma, occurring in 1 to 2 percent of such cases [3]. Approximately 72% of spinal injuries in children under 8 years old occur in the cervical spine [4]. Mechanisms of injury are age related, with younger children sustaining C-spine injuries as a result of motor vehicle-related trauma and adults commonly injured during sporting activities and falls [5]. Spinal column injury may occur in abnormal application of flexion, extension, rotation, compression, distraction and shear forces. Each of these mechanisms causes specific patterns of damage to the bones and ligaments of the spine, wich to some degree translate to patterns of cord damage [6, 7]. C-spine injuries in children generally involve the upper C-spine. The younger the child, the more likely an upper cervical spine injury will occur [8]. But complete lesions of the cord are associated more frequently with lower C-spine injuries [9]. Contrary to what has been reported in the literature, our patient had a trauma of the lower cervical spine. The fact may be explained by unique biomechanics and anatomy of the pediatric cervical spine. They have relatively larger heads than bodies, the position of the cervical spine fulcrum progresses caudally from C2-3 at birth to C5-6 at eight years of age, weaker cervical musculature and increased laxity of the ligaments, immature vertebral joints and horizontally inclined articulating facets that facilitate sliding of the upper cervical spine [10, 11].

Symptoms vary depending on the location of the injury. Spinal cord injury causes weakness and loss of feeling at, and below the injury. How severe symptoms are depends on whether the entire cord is severely injured such as our case. MRI scanning is usually indicated when identification of soft tissue injury is required or if there is a suspicion of spinal cord injury without radiological abnormality [12]. For this reason, we have realized an emergency MRI to the strong suspicion of c-spine cord cut and to avoid the irradiation. Indications for surgery include nonreducible deformities, unstable injuries requiring stabilization, progressive deformity, and decompression of neural structures [13]. Because of the damage caused in the c-spine cord and her hemodynamic instability the patient did not get the surgery. Predictors of mortality include younger age, motor vehicle-related mechanism association with closed head injuries and the highest injury severity score (ISS) [9]. Our patient had two risk factors that explain his death in addition to infectious complications occurred during his stay in intensive care.

## Conclusion

Road accidents are a public health problem in Morocco. It's sad fact that injury is the number one cause of death and disability among children. Anatomical peculiarities as well as differences in the mecanisms explain the preferential localization of injuries of the cervical spine in the child versus adult. The prevention of this scourge involves taking security measures and compliance with the code of the road.
